# Checklist for Habitual Physical Activity (CHaPA) for adults 75 years and older: tool development and content and face validation

**DOI:** 10.1111/psyg.13082

**Published:** 2024-01-29

**Authors:** Kuniko Arita, Yu Ishibashi, Hitomi Ishibashi

**Affiliations:** ^1^ Department of Occupational Therapy, Graduate School of Human Health Science Tokyo Metropolitan University Hachioji Japan; ^2^ Department of Rehabilitation, School of Health Science Tokyo University of Technology Hachioji Japan

**Keywords:** aged, exercise, human activity, validity

## Abstract

**Background:**

Physical activity significantly contributes to older adults' physical and mental health, suggesting that physical activity could enhance healthy life expectancy. Despite its importance for adults aged 75 and older, activity levels among a large proportion of individuals aged 75 and older in Japan do not meet the recommended levels of physical activity, indicating a need for specific strategies tailored to this age group. This study aimed to develop a screening tool that measures daily activities promoting physical activity among older adults and assessed its content and face validity.

**Methods:**

In Stage 1, we derived constructs pertinent to physical activity from previous literature and formulated an item list based on a prior qualitative study we undertook in Japan that evaluated daily behaviours facilitating physical activity among older adults. During Stage 2, we assessed the content and face validity of the list utilising the Nominal Group Technique (NGT), involving eight experts. The content validity was confirmed through two scoring evaluation rounds, while the face validity was verified through the NGT discussion, focusing on the comprehensibility and appropriateness of the tool.

**Results:**

We created a tool with 22 items consisting of three constructs. The NGT participants modified eight of these items for the final assessment, resulting in a finalised tool comprising 22 items that satisfied the adaptation criteria. The content validity of these items was affirmed by median adequacy (>5.0 points) and interquartile range (<1.0 points). The NGT discussion consensus also confirmed satisfactory face validity.

**Conclusion:**

The newly developed tool, Checklist for Habitual Physical Activity (CHaPA) for adults 75 years and older, is a valid screening tool to assess the daily behaviours that facilitate physical activity. This self‐administered instrument aims to assist older adults who need to start and maintain physical activity daily. Before its widespread public deployment, further investigation of the tool's validity and reliability is necessary.

## INTRODUCTION

Physical activity significantly contributes to older adults' physical and mental health, suggesting that activity could enhance healthy life expectancy.^1^ Especially for adults 75 years and older, physical activity is essential for maintaining physical independence and preventing the need for long‐term care. The Japan Geriatrics Society has recommended that the definition of older adults as those aged 65 years and older should be revised to 75 years and older based on differences in physical and living conditions between the contemporary groups aged 65–74 years and those aged 75 and older.^2^ As opposed to those younger than 75 years who are still robust and active,^2^ those aged 75 and older are less likely to engage in physical activity due to changes to their independence.^3^ The incidence of frailty and falls^4^ tends to increase with age and many adults aged 75 and older experience more physical limitations due to chronic diseases.

Regardless of its health benefits, physical activity levels decline with age in most countries, and the rate of older adults who engage in adequate physical activity is stagnant.^5^ The World Health Organization (WHO) Guidelines for Physical Activity and Sedentary Behaviour state that people aged 65 years and older should engage in aerobic physical activity of moderate intensity for at least 150 min or high intensity for at least 75 min per week.^1^ In the world, the percentage of older adults meeting the physical activity guidelines ranged from 2.4% to 83%, with most studies reporting that 20%–60% of the samples met the guidelines.^6^ Studies from other high‐income countries like the United States,^7^ the United Kingdom,^8^ and Australia,^9^ reported that inactivity increases with age among adults aged 75 and older. Similarly in Japan, the rate of older adults who met the physical activity recommendation remains low. The guideline set by the Ministry of Health, Labour, and Welfare (MHLW) emphasises the importance of engaging in physical activity daily, stating ‘at least 40 minutes of physical activity daily, irrespective of intensity.’^10^ In the 2019 National Health and Nutrition Survey, only 42.7% of men and 35.9% of women 70 years and older engaged in physical activity daily, falling short of the national targets of 58% for older men and 48% for older women.^11^

Possible reasons for the low rate of older adults engaging in physical activity include low awareness of the local guidelines, a translational gap between research and practice, and a lack of tools to fill the gap. While guidelines exist to indicate the amount and duration of physical activity, the recognition rate remains low worldwide. In Japan, the guidelines have not been widely disseminated with only 9.3% of adults aged 70 years or older reporting awareness of the MHLW guidelines.^12^ Although there have been few surveys specifically among older adults, studies in the United States,^13^ Canada,^14^ the United Kingdom,^15^ and Australia^16^ pointed out that more understanding of physical activity guidelines should be noted among adults.

While integrating physical activity into daily routines has been pointed out as a promising alternative to structured exercise programs in older adults,^17^ a translation gap remains between evidence and practice^18^ in developing strategies tailored to those aged 75 and older. Barriers to physical activity, especially among older adults, have been identified as health‐related limitations, fear of falling, fatigue,^19^ cognitive burden when using self‐management,^20^ and difficulty in accessing exercise facilities.^21^ Facilitating factors for physical activity among adults aged 60 and older include social interaction, enjoyment, guided activity, and expected health benefits.^21^, ^22^ Baert (2011) noted that most of the previous studies defined adults 60 years of age as older adults and failed to distinguish factors specific to adults 80 years and older. It concluded that barriers to physical activity on the intrapersonal level were similar for younger adults except for fear, which seemed to be a more specific barrier for adults 80 years and older.^19^ Although many studies have reported the factors related to physical activity, some older adults might need a concrete understanding of applying the research results to their daily lives to successfully establish physical activity habits.

While guidelines exist to indicate the amount and duration of physical activity, no tool indicates what specific behaviours should be taken for older adults in later life to achieve these guidelines. In Japan, as long‐term care prevention, screening tools such as the Kihon Check List (KCL)^23^ and Japan Science and Technology Agency Index of Competence (JST‐IC)^24^ are commonly used to identify risks that emerge in daily life, allowing for early detection and early response. However, no screening tool is available to older adults to detect daily behaviours promoting physical activity. Therefore, research needs to investigate the daily behaviours of older adults who successfully overcome the barriers and incorporate the facilitating factors of physical activity into their daily lives to engage in physical activity. This study aimed to develop a screening tool to assess daily behaviours facilitating physical activity for adults 75 years and older and examine its content and face validity.

## METHODS

**Figure 1:**
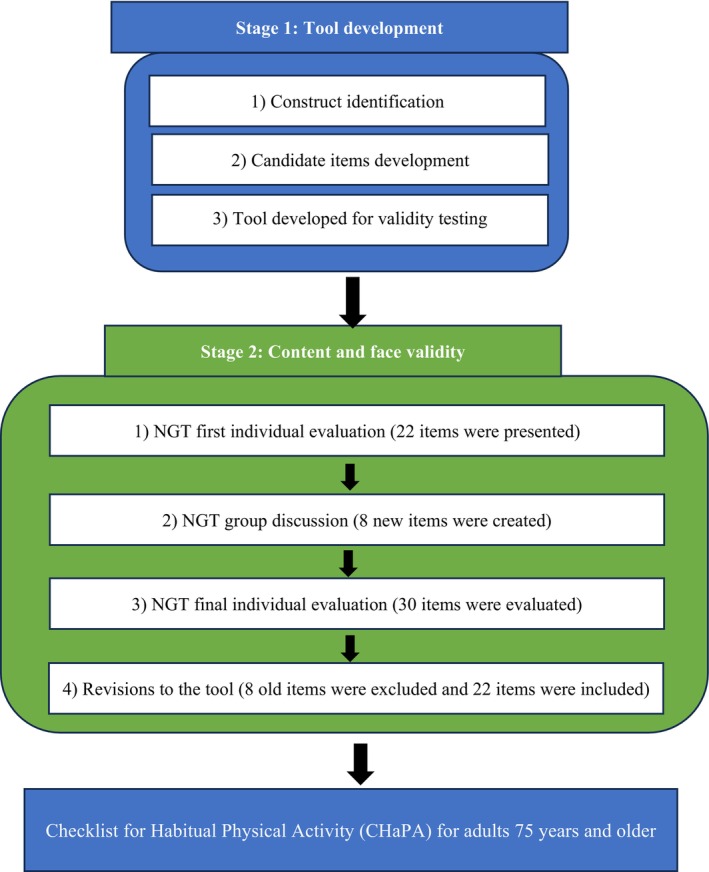
Flow diagram outlining tool development and content and face validity assessment.

We used a methodological research design to develop a tool and assess the tool's content and face validity per COnsensus‐based Standards for the selection of health Measurement INstruments (COSMIN).^25^ We developed the tool in two stages: Stage 1 comprised construct identification and item generation of the tool, and Stage 2 involved testing the content and face validity. Figure 1 illustrates the methods.

### Stage 1: tool development

We followed the two steps of tool item development: identification of constructs and generation of candidate items.^26^

#### Identification of constructs

First, we collected information from previous literature to identify the constructs related to physical activity participation among older adults. We searched for systematic reviews to form the framework of the constructs using the following search terms: physical activity OR exercise, older OR elder, factor OR facilitator* OR barrier* OR motivat* OR determinant* OR correlate* AND review. Following the process, we additionally collected information from the articles describing the constructs of the factors related to older adults' physical activity to understand the constructs further. Moreover, the guidelines and latest updates for physical activity by the WHO,^27^ the MHLW,^28^ and the Japan Sports Agency^29^ were reviewed and analyzed for constructs related to physical activity participation for older adults.

#### Generation of candidate items

To generate tool items, we conducted a qualitative interview study to identify the daily behaviours of active older adults who daily engaged in physical activity. This part of the study is described elsewhere.^30^ In that study, we used the Positive Deviance (PD) method^31^ and extracted 22 behaviour items falling into eight categories. The two participants in the qualitative study reviewed and revised draft constructs and candidate items. The three authors also iteratively reviewed the relevance of the candidate items and the categories to the constructs until they reached an agreement.

### Stage 2: content and face validity

We examined the content and face validity using the Nominal Group Technique (NGT), a method to form a consensus in a group discussion by people with expert knowledge on the issue.^32^ The NGT method was effective when the group members were new to the group, and there was a power imbalance between participants.^33^ The NGT proceeded in the three stages: individual scoring on the candidate items, group discussion regarding the items' face and content validity, and final individual scoring. We assessed content validity by calculating median adequacy and the interquartile range (IQR) at two points: initial and final evaluation. We assessed face validity regarding each item's comprehensiveness and appropriateness using the participants' comments on the first evaluation and verbatim during the NGT group discussion.

#### Participants

NGT participants included those who met either of the following criteria: (i) a medical or welfare‐related worker with experience in supporting older adults; and (ii) adults 75 years and older living in the community who have performed physical activity daily or led physical activity groups. We approached and recruited eight persons to participate in the study using a purposeful sampling method. While groups of between two and 14 participants have been used in nominal group research, at most, seven have been recommended.^32^ In preparation for the sudden absence of the recruited participants due to infectious disease and other reasons, we continued recruiting until eight candidates were recruited.

#### Ethical considerations

We obtained approval from the Ethics Committee of the Tokyo Metropolitan University (Approval No.22058). We assigned IDs to each candidate approached during data collection to enhance the anonymity of the participants. We informed the candidates orally and in writing about the study methods, data confidentiality, voluntary participation, and the right to withdraw without any disadvantage. The candidates became study participants upon verification of their signed consent forms.

#### Content validity

##### Initial evaluation

NGT proceeded in three stages to verify the content validity: initial evaluation, discussion, and final evaluation. The content validation criteria were that the items should be relevant to and cover all the aspects of the constructs that facilitate physical activity (Table 1).

**Table 1:** Constructs and their definitions based on the factors related to physical activity

Constructs	Definitions
Personal level behaviours	Behaviours related to self‐planning, self‐management, health benefit, self‐efficacy, and enjoyment
Intrapersonal level behaviours	Behaviour related to social connection and social support
Community/societal level behaviours	Environmental use in social, built, and natural environment

For the initial evaluation, we distributed a list of items by mail or email. The participants were instructed to score the content validity of each item according to the constructs based on the following five‐point Likert scale: 1, very irrelevant; 2, irrelevant; 3, neither relevant nor irrelevant; 4, reasonably relevant; and 5, relevant. Further space was provided to write additional comments on each item or to suggest specific revisions regarding the relevance to the constructs. All the comments from the initial evaluation are listed in Supporting Information Table S1. After responses were collected, we analyzed the median and IQR of the relevance of each item score. The item content validation criterion was defined as a median adequacy of at least 5.0 and an IQR of less than 1.0, referring to the previous study using NGT.^34^

##### NGT group discussion

Second, all participants joined a group discussion at the research facility. The participants completed a demographic questionnaire as part of the consent process. The facilitator was an occupational therapist with no relation to the study. The facilitator and the researchers had a thorough discussion in advance regarding the proceeding of the NGT discussion. We distributed a list of the comments from the initial evaluation. During the group discussion, the participants collectively discussed the item's relevance to the three constructs. When the participants needed further reference to clarify the meaning of an item, the facilitator referred to the researchers in a separate room and provided the answers to the participants. The discussion was digitally recorded and transcribed verbatim.

##### Final evaluation

After the group discussion, we updated the tool based on the discussion and distributed it to the participants. The participants then scored the final items' relevance to the constructs using the same validation criteria as the initial evaluation. The median and IQR of each item were calculated to confirm a consensus.

#### Face validity

The participants were asked to assess the face validity based on each item's comprehensibility (items should not exceed the literacy level of about a 12‐year‐old, the intention interpreted from a single item should not be complicated, and the length of the item should be appropriate) and appropriateness (items should be relevant to ask adults over the age of 75).^35^ At the first individual evaluation, we asked the participants to comment on each item to suggest specific revisions regarding comprehensibility and appropriateness.

In the group discussion session, the participants suggested revisions to improve the item's comprehensibility and appropriateness for adults aged 75 years and older. When the participants suggested wording changes, the facilitator asked all participants to confirm any wording changes until a final consensus was reached for all items.

## RESULTS

### Stage 1: tool development

#### Identification of constructs

As a result of the literature search, we identified four systematic reviews about the factors related to physical activity,^19^, ^36^, ^37^, ^38^ especially for older adults, and one systematic review^39^ for adults (aged ≧18) and children or adolescents. We also reviewed a systematic review of qualitative studies.^21^ Based on the information we collected from the systematic reviews, we decided to apply the socio‐ecological model,^40^ as shown in the publications by Bauman^39^ and Yarmohammadi^36^ that categorised the factors related to physical activity into the interpersonal, intrapersonal, and environmental levels. The socio‐ecological models enable a macroscopic investigation and provide a more persuasive view of human behaviour.^41^ The ecological models could explain that the physical and social environment were significant determinants of physical activity, and behaviours at each level were not independent but sometimes overlapped and influenced each other.^42^ We integrated the eight categories of behaviours generated from the qualitative study^30^ we conducted in the previous phase into the three constructs (Table 1). The three researchers agreed with the three constructs and the definition, including the eight categories.

#### Generation of candidate items

Two participants aged 75 and older from the qualitative study^30^ reviewed and confirmed the candidate items, categories, and constructs. We developed a screening tool, Checklist for Habitual Physical Activity (CHaPA) for adults 75 years and older. The tool was designed to be self‐administered, and respondents answered ‘Yes’ or ‘No’ to each item based on the implementation in the past month. The total score of the tool was calculated by tallying ‘Yes’ as one point and ‘No’ as zero. The total score ranged from zero to 22. We adopted the answering format from KCL^23^ and JST‐IC^24^ to maintain its simplicity as a screening tool. Since our qualitative study^30^ revealed that some of the respondents had weekly and monthly physical activity habits, we asked them about their daily activities in the past month, with the aim of including monthly‐scheduled physical activity.

### Stage 2: content and face validity

Table 2 describes the NGT participants' characteristics. The participants comprised four healthcare professionals (a public health nurse at a community comprehensive support centre, a physical therapist representing a day service, an occupational therapist at a hospital, and a nurse at a public office) and four older adults. Due to accessibility issues to the research facility, we could not recruit people in their 80s and 90s to participate in a face‐to‐face meeting. Instead, we included participants who led exercise groups or support older adults with disabilities with an understanding of the situation of older adults aged in their 80s and 90s. Four participants were male, and the other four were female. The healthcare professionals were middle‐aged, and older adult participants were 75 years and older. The discussion session lasted for 3 h, with a 45‐min break in the middle. The participants' comments during the discussion are presented in Supporting Information Table S2.

**Table 2:** Nominal Group Technique participant characteristics

ID	Age group	Gender	Years of experience in health care	Years of experience in habitual physical activity
1	70s	F	N/A	30 years
2	70s	M	N/A	15 years
3	70s	F	N/A	10 years
4	70s	M	N/A	15 years
5	30s	F	7 years	N/A
6	20s	M	5 years	N/A
7	50s	F	35 years	N/A
8	30s	M	8 years	N/A

Abbreviation: N/A, not applicable.

#### Content validity

Table 3 shows the results of the initial and final evaluation. Table 4 indicates the final version of the CHaPA. The number of items was 22 for the initial evaluation, and the participants created eight revised items during the discussion, bringing the total to 30 items. Finally, 23 items met the content validation criteria for adoption. We adopted a modified item if the original and modified items that stated the same contents met the criteria, resulting in 22 final items.

**Table 3:** Items and their medians and interquartile ranges

Number	Items	Initial evaluation							Final evaluation						
		Number of persons					Median	IQR	Number of persons					Median	IQR
		1	2	3	4	5			1	2	3	4	5		
①	Timeframe for physical activity is determined on a daily, weekly, or monthly basis	0	0	1	1	6	5	0.25	0	0	0	0	8	5	0
②	Waking up by 7:00 a.m.	0	1	1	5	1	4	0.25	1	1	2	1	3	3.5	2.25
②‐a	Waking up at a set time								0	0	0	3	5	5	1
③	Walking to shopping	0	0	2	2	4	4.5	1.25	0	1	2	2	3	4	2
③‐a	Maintaining a habit of walking to the store								0	0	1	1	6	5	0.25
④	Using units that are easy to understand (metres, minutes, steps) to keep track of activity level	0	1	0	1	6	5	0.25	0	0	0	0	8	5	0
⑤	Tracking activity needed when travelling to neighbourhood landmarks (e.g., bus stops, supermarkets)	0	0	1	1	6	5	0.25	0	0	0	1	7	5	0
⑥	Engaging in physical activity to the point of feeling tired	0	0	2	2	4	4.5	1.25	0	0	0	0	8	5	0
⑦	Regularly measuring values related to your physical status (e.g., weight, blood pressure, body fat)	0	0	1	0	7	5	0	0	0	0	0	8	5	0
⑧	Using body activities with an awareness of which parts of the body they benefit	0	0	2	2	4	4.5	1.25	0	0	1	2	5	5	1
⑧‐a	Being aware of which parts of the body are affected by your physical activity								0	0	0	0	8	5	0
⑨	Completing the parts you can do, regardless of the level of accomplishment	0	1	2	3	2	4	1.25	0	0	0	1	7	5	0
⑩	Doing physical activity according to your standards, even if different from the national guideline	1	0	2	1	4	4.5	2	1	0	1	4	2	4	0.5
⑩‐a	Doing physical activity according to your own standards, even if different from the national guideline*								0	0	0	1	7	5	0
⑪	Incorporating movements from other's and media information related to physical activity	0	1	1	0	6	5	0.5	0	0	0	0	8	5	0
⑫	Doing familiar physical activities (things you did when you were young, when you were a child)	0	0	1	3	4	4.5	1	0	0	0	6	2	4	0.25
⑫‐a	Doing familiar physical activities (e.g., things you did when you were young, when you were a child)								0	0	0	0	8	5	0
⑬	Getting daily enjoyment other than exercise (e.g., meeting people, getting close to nature, observing the environment) during physical activity	0	0	1	1	6	5	0.25	0	0	1	3	4	4.5	1
⑬‐a	Getting daily enjoyment out of physical activity (e.g., meeting people, getting close to nature, observing the environment)								0	0	0	0	8	5	0
⑭	Having a purpose other than exercise (e.g., looking after the community, visiting friends) for your physical activity	0	0	1	5	2	4	0.25	0	0	0	2	6	5	0.25
⑮	Talking to others during physical activity	0	0	0	2	6	5	0.25	0	0	0	1	7	5	0
⑯	Doing physical activity in the presence of people older than yourself	0	1	2	3	2	4	1.25	0	0	0	2	6	5	0.25
⑰	Having a group role when doing physical activity with others (e.g., preparing and cleaning up, taking care of people and pets, managing equipment)	0	0	0	2	6	5	0.25	0	0	0	0	8	5	0
⑱	Talking to family and friends about the physical activity you are doing	0	0	1	0	7	5	0	0	0	0	0	8	5	0
⑲	Talking to your family doctor about the physical activity you are doing	0	0	1	0	7	5	0	0	0	0	1	7	5	0
⑳	Setting walking courses taking into account the local environment (e.g., crime prevention, safety, restrooms, resting places)	0	0	1	1	6	5	0.25	0	0	0	0	8	5	0
㉑	Using buses in combination when you are tired or have luggage	0	0	2	1	5	5	1.25	0	0	2	3	3	4	1.25
㉑‐a	Using public transportation (e.g., buses) in combination when you are tired or have luggage								0	0	0	0	8	5	0
㉒	Doing physical activity in the places you usually pass	0	0	3	2	3	4	2	0	0	3	4	1	4	1
㉒‐a	Regularly utilising nearby stairs, slopes for physical activity								0	0	0	0	8	5	0
	IRQ, interquartile range														

**Table 4:** Checklist for Habitual Physical Activity for people aged 75 years and older

	Response	
Securing physical activity time		
① Timeframe for physical activity is determined on a daily, weekly, or monthly basis	Yes	No
② Waking up at a set time	Yes	No
③ Maintaining a habit of walking to the store	Yes	No
Monitoring amount of physical activity		
④ Using units that are easy to understand (metres, minutes, steps) to keep track of activity level	Yes	No
⑤ Tracking activity needed when travelling to neighbourhood landmarks (e.g., bus stops, supermarkets)	Yes	No
⑥ Engaging in physical activity to the point of feeling tired	Yes	No
Having a way to feel the effects of activity on your body		
⑦ Regularly measuring values related to your physical status (e.g., weight, blood pressure, body fat)	Yes	No
⑧ Being aware of which parts of the body are affected by your physical activity	Yes	No
Adopting your own unique activity regimen		
⑨ Completing the parts you can do, regardless of the level of accomplishment	Yes	No
⑩ Doing physical activity according to your standards, even if different from the national guidelines*	Yes	No
⑪ Incorporating movements from other's and media information related to physical activity	Yes	No
⑫ Doing familiar physical activities (e.g., things you did when you were young, when you were a child)	Yes	No
Having fun/purpose other than exercise during physical activity		
⑬ Getting daily enjoyment out of physical activity (e.g., meeting people, getting close to nature, observing the environment)	Yes	No
⑭ Having a purpose other than exercise (e.g., looking after the community, visiting friends) for your physical activity	Yes	No
Interacting with others as a part of physical activity		
⑮ Talking to others during physical activity	Yes	No
⑯ Doing physical activity in the presence of people older than yourself	Yes	No
⑰ Having a group role when doing physical activity with others (e.g., preparing and cleaning up, taking care of people and pets, managing equipment)	Yes	No
Sharing with others about your physical activities		
⑱ Talking to family and friends about the physical activity you are doing	Yes	No
⑲ Talking to your family doctor about the physical activity you are doing	Yes	No
Utilising local environmental resources		
⑳ Setting walking courses taking into account the local environment (e.g., crime prevention, safety, restrooms, resting places)	Yes	No
㉑ Using public transportation (e.g., buses) in combination when you are tired or have luggage	Yes	No
㉒ Regularly utilising nearby stairs, slopes for physical activity	Yes	No

*Note*: **Physical activity** is any movement that consumes more energy than the resting state. Physical activity includes both **‘exercise’** and **‘activities of daily living’**. **Exercise:** among physical activities, those that are planned, deliberate, and sustained for the purpose of maintaining or improving physical fitness. (Examples of exercise: training and aerobics at a gym or fitness club; sports such as tennis, soccer, and basketball; leisure time walks and active hobbies). **Activities of daily living:** among physical activities, daily labour, housework, commuting to and from work. (Examples of activities of daily living: household chores such as shopping and hanging laundry to dry, daily activities such as walking the dog and playing outdoors with children, and work‐related activities such as commuting to work, making sales trips outside, climbing stairs, carrying packages, farming, and fishing activities”). The national guidelines*: At least 40 minutes of physical activity per day, regardless of the intensity, for older adults over 65 years (Ministry of Health, Labour, and Welfare: 2013).

In the initial evaluation, 11 candidate items (1, 4, 5, 7, 11, 13, 15, 17–20) met the content validation criteria for adoption. Ten candidate items did not meet the content validation criteria for adoption (2, 3, 6, 8–10, 12, 14, 16, 22), having a median value of less than 5.0, and seven items (3, 6, 8–10, 16, 21) had an interquartile range larger than 1.0.

At the final evaluation, 23 items met the content validation criteria for adoption. The three items cleared the content validation criteria and were retained from the original items (7, 18, 19). Four candidate items (6, 9, 14, 16) did not meet the criteria at the first evaluation but met the content validation criteria for adoption without any modifications. The participants revised eight items (2, 3, 8, 10, 12, 13, 21, 22) and created eight new items (2‐a, 3‐a, 8‐a, 10‐a, 12‐a, 13‐a, 21‐a, 22‐a), and they reached the content validation criteria at the final evaluation. The original item 8 and revised item 8‐a met the content validation criteria, but we adopted the 8‐a as the final item, reflecting the NGT discussion. We deleted items that did not meet the adaptation criteria at the final evaluation (2, 3, 10, 12, 13, 21, 22).

#### Face validity

The major suggestions for revisions in response to the NGT included revising the definition of physical activity and adding the national physical activity guideline. Two participants suggested revising the definition of physical activity on the tool because older adults did not commonly understand the definition. At the time of the initial assessment, physical activity was defined as any movement that consumes more energy than the resting state, including daily activities as well as exercise. However, the participants recognised that eating and toileting could also be included in the physical activity. In response to a suggestion that the definition of physical activity was not common knowledge, we changed the original definition to a more detailed one, referring to the definition provided by the MHLW.^43^ The revised definition was added to Table 4: Physical activity is any movement that consumes more energy than the resting state. Physical activity includes both ‘exercise’ and ‘activities of daily living.’ Exercise: among physical activities, those that are planned, deliberate, and sustained for the purpose of maintaining or improving physical fitness. Examples of exercise: training and aerobics at a gym or fitness club; sports such as tennis, soccer, and basketball; leisure time walks and active hobbies. Activities of daily living: among physical activities, daily labour, housework, commuting to and from work. Examples of activity of daily living: household chores such as shopping and hanging laundry to dry, daily activities such as walking the dog and playing outdoors with children, and work‐related activities such as commuting to work, making sales trips outside, climbing stairs, carrying packages, farming, and fishing activities.^43^

The participants also suggested explaining the physical activity guidelines because the participants stated that the national guidelines were not common knowledge among targeted older adults. For example, item 10 was revised to ‘10‐a: Doing physical activity according to your standards, even if different from national guideline* (guideline* is provided as footnotes at the bottom of the form).’

## DISCUSSION

The study describes the development and validity assessment of a tool that screens the daily behaviours facilitating physical activity among adults 75 years and older. The NGT method ensured tool items' content validity and face validity for the targeted age group by reflecting the lifestyle of adults aged 75 and older. The CHaPA could be a practical screening tool that meets a need and bridges a translation gap in the evidence and practice for facilitating physical activity of the age group.

### Content and face validity

The NGT process verified that CHaPA covered eight categories within the three constructs: behaviours at the personal, intrapersonal, and environmental levels. As suggested by Lee (2021), a macroscopic understanding is needed to explain individual levels of physical activity,^41^ the tool screens daily behaviours that occur at the three levels, including intrapersonal and environmental. The items, which reflect daily behaviours relevant to the adults aged 75 and older, showed consistently high content validity, with a median of 5 and an IQR of 0 from the first to the final assessment. For example, item ‘7: Regularly measuring values related to your physical status (e.g., weight, blood pressure, body fat)’ is one of the methods used to recognise the health benefits of physical activity. Although recognising the health benefits of physical activity is considered a facilitating factor in general,^39^ no previous study has provided specific examples of how to recognise the benefits of physical activity. The item introduced the behaviour that adults aged 75 and older could practise relatively easily, such as weight measurement at home, regular blood pressure measurements at their family doctors, and measurements of body fat, muscle mass, and grip strength at exercise classes.

The items at the intrapersonal level also quickly reached a consensus because the items were relevant to the older adults' standard status of social connection and did not require building new social interactions. The items with consistently high consensus included ‘18: Talking to family and friends about the physical activity you are doing’ and ‘19: Talking to your family doctor about the physical activity you are doing.’ Social support and connections have proven to motivate older adults' participation in physical activity.^37^ The items provided examples of how older adults could obtain social support in their community without trying to expand new social networks.

The four items that did not meet the content validity criteria in the initial evaluation but met the criteria in the final evaluation without any modifications include: ‘6: Engaging in physical activity to the point of feeling tired,’ ‘9: Completing the parts you can do, regardless of the level of accomplishment,’ ‘14: Having a purpose other than exercise (e.g., looking after the community, visiting friends) for your physical activity,’ and ‘16: Doing physical activity in the presence of people older than yourself.’ Some of the NGT participants had difficulties understanding if the items were properly related to the constructs when individually scoring the items. However, the NGT discussion processes could have improved their understanding of the items' relations to the constructs. Since different interpretations might have existed among the participants, further validation of the content would be necessary. Because the level of understanding of these items might vary among individuals, consideration would be required, such as having the screening conducted in a group setting or having a support person add explanations.

Eight revised items met the criteria for content validity after revisions. The NGT process improved appropriateness for the target populations, simultaneously improving its face validity. The modifications could be categorised into three types: (i) correction of unclear words; (ii) generalisation of the items' content; and (iii) clarification by changing the syntax. For example, as the modification category (i) specific correction of unclear words, ‘22: Doing physical activity in the places you usually pass’ was modified to ‘22‐a: Regularly utilising nearby stairs, slopes for physical activity.’ The modification cleared the item by identifying the specific environmental resources the older adults could utilise as a place for physical activity. For example, as the modification category (ii) generalisation of the items' content, ‘2: Waking up by 7:00 a.m.’ was modified to ‘2‐a Waking up at a set time’. ‘3: Walking to shopping’ was modified to ‘3‐a: Maintaining a habit of walking to the store.’ In addition, the word *exempli gratia* (e.g.) was added to items 12 and 21 to encompass the various lifestyles of the older adults. For example, the original item 21 meant using transportation limited to buses, but the final item 21 indicated the use of buses and other types of public transportation. As the modification category (iii) clarification by changing the syntax, ‘8: Using physical activities with an awareness of which parts of the body they benefit’ was modified to ‘8‐a: Being aware of which parts of the body are affected by your physical activity’. ‘13: Getting daily enjoyment other than exercise (e.g., meeting people, getting close to nature, observing the environment) during physical activity’ was also modified to ‘13‐a: Getting daily enjoyment out of physical activity (e.g., meeting people, getting close to nature, observing the environment)’ by changing the syntax. The tool became appropriate for the target population by keeping the syntax simple.

The face validity of the tool was adequately verified due to the following reasons. First, clarification of the definition of physical activity and revision of the national guideline were necessary changes to improve the comprehensibility of the tool. Second, the final version of the tool reflected the opinions of both the target population, 75 years and older and the healthcare professionals who have experience in assisting with older adults' health.

### Characteristics of the adopted items

The adopted items included behaviours that overcome barriers for physical activity specific to older adults: self‐management burden,^20^ fear of falling and injuries,^19^ and access difficulties to exercise facilities.^21^ Although older adults are more likely to feel burdened by self‐management, such as keeping track of the amount of exercise they do,^20^ ‘securing physical activity time’ and ‘monitoring the amount of physical activity’ categories provided examples of overcoming the sense of burden. The ‘adopting your own unique activity regimen’ category listed behaviours to only engage in physical activities appropriate for their health status and physical abilities. In addition, while difficulty accessing exercise facilities was a barrier, the items in the ‘utilising local environmental resources’ category provided tips on using environmental resources that already exist within the community where the older adults reside.

The adopted items also suggest incorporating facilitating factors specific to older adults into daily life: health benefits and enjoyment during physical activity.^22^ The items in the ‘having a way to feel the effects of activity on your body’ indicated how to recognise the health benefits at home and within the older adults' living community. The ‘having fun /purpose other than exercise during physical activity’ category provided examples of enjoyment derived through physical activity, even if the exercise itself was not enjoyable. The tool can be used as a screening and a reference tool to check the behaviours that can be incorporated into one's life.

The CHaPA was developed based on the PD method, which improves community health by identifying and disseminating behaviours that have positive outcomes and are practicable by people within the same environment without using external resources. Therefore, the CHaPA included behaviours that older adults could easily practice without specific resources or funding. In this respect, the CHaPA differed in its purpose from the existing screening tool, such as KCL^23^ and JST‐IC.^24^ KCL aims to check for any areas of decline in the physical and mental functions of adults aged 65 and older. JST‐IC has been developed as a scale to measure the abilities necessary for independent and active living. It asks whether a person can perform a particular activity but does not suggest tips to support that ability. The CHaPA, on the other hand, provides concrete behavioural examples that can be incorporated into daily lives among older adults to improve their participation in physical activity.

### Limitations of the study and potential use of the tool

The study has some limitations. First, we conducted NGT only once, and all the opinions were not discussed and reflected. Although the tool's basic level of content and face validity have been investigated, construct validity, criterion‐related validity, and re‐test reliability must be examined before public use. Second, because the tool was created based on Japanese older adults' lifestyles, the generalisability outside Japan may be limited. However, the tool can promote physical activity from the aspect of daily behaviours among adults 75 years and older because it provides concrete examples easily incorporated into their daily lives. Once cultural relevance is verified, the screening tool can be utilised in countries with a culture similar to Japan's. Also, the method we used to develop a new tool can be applied to address other public health issues. Although the tool was designed for people 75 and older, it can also be applicable for adults under 75 years of age as a preliminary reference, for example, behaviours to maintain their physical activity level in the future.

#### CONCLUSIONS

The study describes the development and validation the CHaPA to screen daily behaviours that facilitate physical activity among adults 75 years and older. The content validity of CHaPA was verified as the NGT evaluations reached median adequacy and the IQR. The opinions of the target population, 75 years and older, and the healthcare professionals, have contributed to establishing acceptable face validity of the tool. Before its widespread public deployment, further investigation of the tool's validity and reliability is necessary.

## AUTHOR CONTRIBUTIONS

**Kuniko Arita:** funding acquisition, conceptualisation, methodology, investigation, formal analysis, writing – original draft preparation. **Yu Ishibashi**: formal analysis, supervision, data curation. **Hitomi Ishibashi:** formal analysis, writing – review and editing.

## DISCLOSURE

There are no potential conflicts of interest to declare.

## ETHICS APPROVAL STATEMENT

We obtained approval from the Ethics Committee of the Tokyo Metropolitan University (Approval No.22058).

## PATIENT CONSENT STATEMENT

We informed the candidates orally and in writing about the study methods, data confidentiality, voluntary participation, and the right to withdraw without any disadvantage. Study candidates became study participants upon submission of a consent form.

## Supporting information


**Table S1.** Initial evaluation comments for content and face validity.

**Table S2.** Comments for content validity and face validity.

## Data Availability

The data that support the findings of this study are available from the corresponding author upon reasonable request.
