# Supplementary material for: Checklist for Habitual Physical Activity (CHaPA) for adults 75 years and older: tool development and content and face validation
Source: Psychogeriatrics. 2024 Jan 29;24(2):355–68. doi: 10.1111/psyg.13082 (PMC11578024; doi:10.1111/psyg.13082)
Supplement: Supplementary file 1 — Table S1. Initial evaluation comments for content and face validity. [file PSYG-24-355-s002.docx]

|  | Supporting Information Table 1: Initial evaluation comments for content and face validity |
| --- | --- |
| ① | **Timeframe for physical activity is determined on a daily, weekly, or monthly basis** |
|  | Revise the definition of Physical activity (e.g., XX, etc.) （ID8） |
|  | Which is it, what time of day is it, or is it one hour a day, etc.? I thought it would be perceived differently by different people.　（ID5） |
|  | Shows planning for physical activity（ID6） |
| ② | **Waking up by 7:00 a.m.** |
|  | “I'm up by seven in the morning."（ID8） |
|  | I didn't know what it meant to get up by 7:00 a.m.（ID7） |
|  | What is the rationale for 7:00?（ID5） |
|  | Older people are assumed to be early risers.　（ID1） |
| ③ | **Walking to shopping** |
|  | “I walk to the store to shop.”（ID8） |
|  | Since men don't seem to shop, I thought there was no need to limit it.（ID5） |
|  | I know if you are in the habit of walking, but the distance is ambiguous.（ID1） |
| ④ | **Using units that are easy to understand (meters, minutes, steps) to keep track of activity level** |
|  | I think you can understand the minutes, but you can't meter or steps.（ID7） |
|  | Pedometer, smart phone, top watch ours will tell us.（ID1） |
| ⑤ | **Tracking activity needed when traveling to neighborhood landmarks (e.g. bus stops, supermarkets)** |
|  | Can you determine the specific amount of activity?（ID7） |
|  | Able to track daily activity（ID1） |
| ⑥ | **Engaging in physical activity to the point of feeling tired** |
|  | Physical activity → Activity amount（ID8） |
|  | Since the definition of tiredness varies from person to person, I think it would be better to be a little more specific, such as "to the extent that you get a little out of breath. (ID5) |
|  | Sense of fatigue is in balance with other activities. Can the sense of fatigue from physical activity alone be ascertained?（ID1） |
| ⑦ | **Regularly measuring values related to your physical status (e.g. weight, blood pressure, body fat)** |
|  | Few people even measure body fat.（ID7） |
|  | It would be better if muscle mass were included. But some people can't measure it.（ID1） |
| ⑧ | **Using body activities with an awareness of which parts of the body they benefit** |
|  | I do wish they would be aware of it.（ID7） |
|  | The predicate "I am using" seemed a bit unnatural for the subject "physical activity is -" e.g.: I am aware of which part of the body physical activity has an effect on.（ID6） |
|  | I use" (tool name ex. grips, weights) or "I do" (tool name ex. grips, weights)（ID2） |
|  | When physical activity becomes habitual, we may use it without even being aware of it.　（ID1） |
| ⑨ | **Completing the parts you can do, regardless of the level of accomplishment** |
|  | That would be considered a good thing.（ID7） |
|  | I found it somewhat difficult to understand "only the part I can do -" e.g., I only do the part I deem I can do, regardless of the level of achievement.（ID6） |
|  | I'm doing the parts I can in the ways I can."（ID6） |
| ⑩ | **Doing physical activity according to your standards, even if different from the national guideline** |
|  | Are you familiar with the guidelines?（ID7） |
|  | National Guidelines" Appendix listed or deleted.（ID2） |
|  | What are the national guidelines? I know some of you may be wondering, "What are the national guidelines?（ID1） |
| ⑪ | **Incorporating movements from other’s and media information related to physical activity** |
|  |  |
| ⑫ | **Doing familiar physical activities (things you did when you were young, when you were a child)** |
|  | Proposed amendment: "I am still doing the physical activities I did when I was younger and younger."（ID1） |
| ⑬ | **Getting daily enjoyment other than exercise (e.g., meeting people, getting close to nature, observing the environment) during physical activity** |
|  | I found it difficult to understand the difference between 13 and 14, e.g. 13 (meeting someone) and 14 (going to see a friend).（ID6） |
|  | Added "Enjoy the Conversation"（ID2） |
|  | Having something to look forward to increases motivation for physical activity.（ID1） |
| ⑭ | **Having a purpose other than exercise (e.g., looking after the community, visiting friends) for your physical activity** |
|  | The objective is primary and the physical activity is secondary, but if the results are good, then it's good enough.　（ID1） |
| ⑮ | **Talking to others during physical activity** |
|  | I am concerned about the level of conversation. Can it be as little as a greeting?（ID1） |
| ⑯ | **Doing physical activity in the presence of people older than yourself** |
|  | Do you know the ages of others?（ID7） |
|  | What is the purpose of asking this?（ID5） |
|  | Items you want to know if the elderly are in the mix?（ID1） |
| ⑰ | **Having a group role when doing physical activity with others (e.g., preparing and cleaning up, taking care of people and pets, managing equipment)** |
|  | I imagine a team or group, is that correct? It is hard to convey the meaning of taking care of people and pets.（ID1） |
| ⑱ | **Talking to family about the physical activity you are doing** |
|  | Since some people live alone, it would be nice to include friends.（ID5） |
|  | How about as a single person talking to a family member or person（ID1） |
| ⑲ | **Talking to your family doctor about the physical activity you are doing** |
|  | In the case of orthopedics, it is a direct link, but in other departments, I don't think there are many opportunities to talk about it.（ID1） |
| ⑳ | **Setting walking courses taking into account the local environment (e.g. crime prevention, safety, restrooms, resting places)** |
|  | On the other hand, who can walk around without thinking about the local environment?（ID5） |
| ㉑ | **Using buses in combination when you are tired or have luggage** |
|  | Better to use buses, etc. (Metropolitan Electric Railway)（ID7） |
|  | Changed to "use of buses or other non-walking means of transportation."（ID5） |
| ㉒ | **Doing physical activity in the places you usually pass** |
|  | Even if you don't pass through it, if it is within your daily life, you can say that you are utilizing the resources of the community.（ID5） |
|  | It depends on the environment in which you are placed.（ID1） |
