# Supplementary material for: Checklist for Habitual Physical Activity (CHaPA) for adults 75 years and older: tool development and content and face validation
Source: Psychogeriatrics. 2024 Jan 29;24(2):355–68. doi: 10.1111/psyg.13082 (PMC11578024; doi:10.1111/psyg.13082)
Supplement: Supplementary file 2 — Table S2. Comments for content validity and face validity. [file PSYG-24-355-s001.docx]

Supporting Information_ Table 2: Comments for Content validity and Face validity

| ＃ | Item |
| --- | --- |
| ① | **Timeframe for physical activity is determined on a daily, weekly, or monthly basis** |
|  | I think it is very important to have a time frame at the very beginning, and to use it clearly at the beginning for personal planning and self-management, which is what we call self-planning and self-management here.（ID1） |
|  | I am sure there will be differences in the way each person perceives the situation. I think it is important to listen to the first question in order to be conscious of the time.（ID2） |
|  | Something about that timeframe and the time of day, so rather than a timeframe, I'd rather have that kind of action goal action.（ID2） |
|  | Time is a big part of it, and this could be good in terms of making people aware of things like that time schedule.（ID1） |
|  | In terms of physical activity, if we look at this terminology, it includes activities of daily living, such as taking a meal at what time during the day, eating something at this time, and taking a bath. It is a little difficult to understand unless we get the definitions right.（ID8） |
| ② | **Waking up by 7:00 a.m.** |
|  | I think that getting up early in the morning might be one of the implications of the health habit, but I'm not sure what you mean by that.（ID1） |
|  | I think you sleep until about 7:00 a.m. when you are not feeling well, but if you are in good health, I don't think there are many really elderly people who can sleep until 7:00 a.m.（ID3） |
|  | I know many of the people I work with are less energetic than you are, so I wake up quite a few of them, but then they go back to sleep and stay asleep until about 9:00.（ID7） |
|  | I'm not really sure what you mean by being up by 7:00 a.m.（ID4） |
|  | It's rather easy to understand if it's "I have a set wake-up time.　（ID2） |
|  | Might as well make it a "set time." (ID１) |
| ③ | **Walking to shopping** |
|  | Health benefits, I mean, health benefits, in the sense that people go out of their way to go shopping on foot, even though it would be better to walk or ride a bicycle because it is good for their health.（ID1） |
|  | I think there are people who feel that they can do it right in front of them, so for those people, we need to do something about the fact that they can do it on foot.（ID4）。 |
|  | Or, "I'll walk to the store, even if it's a little far."　(ID4) |
|  | I think you should add a note to the part about walking to shopping, whether it's distance or time. (ID7) |
|  | One proposed change: "I'm in the habit of walking to the store."　(ID1) |
| ④ | **Using units that are easy to understand (meters, minutes, steps) to keep track of activity level** |
|  | As a construct, number 4 still falls in the area of self-management.　（ID6） |
|  | Number 4 is more specific. This is the question of what unit of measure is used to determine the amount of both activities.（ID4） |
|  | One example is a pedometer, which is attached to my current cell phone, so I think using a cell phone to measure is one method. I think this activity amount is also one way to measure the number of steps. I think it is one way to measure the amount of activity. This is a long story, but this is the core of what we mean when we say, for example, how many meters, how many minutes, and how much.（ID5） |
|  | For example, in the case of number 5, after answering number 4, you will probably answer number 5, which asks whether you are replacing the unit of activity with a unit that is easy for you to understand. After answering this question, number 5, the amount of activity, means that if you yourself measure the amount of activity in meters, for example, in number 4, then in number 5, it means whether you know the meters to the bus stop supermarket, etc.（ID1） |
|  | However, I don't know my activity level at all.（ID5） |
|  | If I were to put parentheses on all of them, again, maybe there will be future items that I will have to put on, maybe.（ID2） |
|  | This time, the number 5 is supplemented by an example of a bus stop supermarket as a landmark, so I guess that's the point.（ID2） |
| ⑤ | **Tracking activity needed when traveling to neighborhood landmarks (e.g. bus stops, supermarkets)** |
|  | It's hard to tell the range of activity.　(ID4)。 |
|  | For example, how many meters, how many minutes, how much, etc., is self-discipline, and this is the core. (ID1) |
|  | After answering the question 4, I am sure that you will be able to answer number 5, which asks whether you are replacing the unit of activity with a unit that is easy for you to understand. After answering this question, number 5, the amount of activity, means that if you yourself measure the amount of activity in meters, for example, in number 4, it means whether or not you know the meters to the bus stop supermarket, etc. in number 5. But I don't know my activity level at all. (ID2) |
|  | I think the fourth item is the one that shows the amount of activity as a unit, so it would seem that the definition of the amount of activity is different.（ID1） |
|  | I'm fine with it as it is. Yes, I thought. It is only a section. From home to the supermarket or from home to the bus stop, there are only these three things anymore when it comes to the amount of activity. Yes, no, I don't need to repeat them politely anymore, I don't think.。(ID2) |
| ⑥ | **Engaging in physical activity to the point of feeling tired** |
|  | Is it okay for everyone here to say that they feel tired of that standard separately? (ID2)。 |
|  | The definition of exhausting is too different from person to person to evaluate. (ID5) |
|  | I know what you mean by self-discipline, working up to the point of feeling tired, depending on how tired each of us is.（ID6） |
| ⑦ | **Regularly measuring values related to your physical status (e.g. weight, blood pressure, body fat)** |
|  | I think the concept of health benefits is all expressed right here, right now. (ID1) |
|  | Very easy to understand, I record my weight, blood pressure, and body fat daily.（ID2） |
| ⑧ | **Using body activities with an awareness of which parts of the body they benefit** |
|  | That's easy to understand as a concept. (ID3) |
|  | Hard to understand the expression "using".（ID8） |
|  | It may be a little strange to say that they are using it because it is the subject of physical activity.（ID3） |
|  | How about "I am aware of which parts of my body physical activity has an effect on."（ID6） |
| ⑨ | **Completing the parts you can do, regardless of the level of accomplishment** |
|  | It's strikingly pithy.（ID4） |
|  | Is "doing" something official in Japanese?（ID5） |
|  | I was wondering if they would use the spoken language in such a formal document like this, but if it is easy to understand, it might be a good idea.（ID5） |
|  | I'm working on it, but it's important by implication.（ID2） |
|  | You don't have to force the connections to align.　（ID3） |
|  | I think it's fine to say "I do it in everyday conversation" because it's an easy-to-understand expression.（ID2） |
| ⑩ | **Doing physical activity according to your standards, even if different from the national guideline** |
|  | And I wonder if the guideline is an easy-to-understand expression, or if there should be an annotation in the guideline.（ID7） |
|  | You have a very wide range of guidelines. So it is absolutely necessary to change it. Should be（ID1） |
|  | 'I do physical activity according to my own standards, regardless of the standards of others.'（ID3） |
|  | Since one's own standard is a matter of course, I think it is necessary to compare one's own standard with the previous standard, and in that way, the previous standard is necessary in order to show the standard.（ID2） |
|  | The word "standards" should be used in such a way that the standards move in circles, so the guidelines of the country should be included in the wording.（ID1） |
|  | For example, it would be odd to remove just this guideline, so the proposed change is "I am physically active according to my own standards."（ID4） |
|  | It is the love of the person who reads it or does not read it, so I think it is better to just annotate it after all.（ID7） |
| ⑪ | **Incorporating movements from other’s and media information related to physical activity** |
|  | I'm not sure about the self-efficacy part.（ID8） |
|  | I think that's a good statement.（ID5） |
|  | Personally, I would like to try what I have seen on TV after reading this text. So, unexpectedly, when I hear on TV that something like this is good, I think, "Let's try it.（ID2） |
| ⑫ | **Doing familiar physical activities (things you did when you were young, when you were a child)** |
|  | It's self-efficacy in the sense that I can do it because I did it when I was little.（ID6） |
|  | The fact that the expression "when I was small" is included in the phrase "when I was young" limits it to just that. It could include a bit later. It would be different if the word "etc." was used only after the word "etc.". Then it is not limited.（ID1） |
|  | It looks very natural. I think it's good.（ID4） |
| ⑬ | **Getting daily enjoyment other than exercise (e.g., meeting people, getting close to nature, observing the environment) during physical activity** |
|  | It is in the construct of enjoyment.（ID4） |
|  | I'm a little concerned about the "non-exercise" part of physical activity. I think it is so important that it is almost always used in the second half of this article. I think it is such an important word.（ID8） |
|  | I think it's okay to remove it except for exercise.（ID4）（ID8） |
|  | I'm quite excited to meet people.（ID3） |
|  | I feel that the "exercise" part could be made a little more clear.（ID7） |
| ⑭ | **Having a purpose other than exercise (e.g., looking after the community, visiting friends) for your physical activity** |
|  | If you remove the first half part, it would emphasize the back more.（ID6） |
|  | Difficult to understand. Lengthy.（ID7） |
|  | I think this sentence next time can be left as it is for purposes that are not exercise during physical activity（ID3） |
| ⑮ | **Talking to others during physical activity** |
|  | I think that there are many meanings to meeting one's friends, even by chance. Yes, it does. It is very important to have a conversation there, isn't it?（ID1） |
|  | It will be easy to understand as a sentence.（ID4） |
|  | I think it is a good compositional concept.（ID5） |
| ⑯ | **Doing physical activity in the presence of people older than yourself** |
|  | Understanding seems fine.（ID3） |
|  | It's also good in terms of social connections, isn't it?（ID4） |
|  | I think it's very easy to understand and convey the message in a way that's very clear and expressive.（ID4） |
| ⑰ | **Having a group role when doing physical activity with others (e.g., preparing and cleaning up, taking care of people and pets, managing equipment)** |
|  | This is also easy to understand.（ID2） |
|  | So I think this is a good idea because it's about relationships with people and social connections.（ID1） |
| ⑱ | **Talking to family about the physical activity you are doing** |
|  | Social support for the constructs is also not a problem.（ID4） |
|  | It is easy to understand.（ID3） |
| ⑲ | **Talking to your family doctor about the physical activity you are doing** |
|  | You can also visualize it concretely. Yes, number 19 is easy to understand.（ID3） |
|  | Sometimes when I talk about it, on the contrary, people tell me I shouldn't do it...it's social support.（ID2） |
| ⑳ | **Setting walking courses taking into account the local environment (e.g. crime prevention, safety, restrooms, resting places)** |
|  | I am addicted to the concept of configuration. Yes, I am. No problem.（ID8） |
|  | It's going to take you a while to figure it out.（ID5） |
|  | Is the term "walk course" easy to understand?（ID5） |
|  | But as you get older, the walking trails are easier to follow. I think it is very natural.（ID1） |
| ㉑ | **Using buses in combination when you are tired or have luggage** |
|  | Clear in terms of the use of local resources　(ID3) |
|  | That they dare to use only buses. It could be something like coming back on the TEPCO.（ID7） |
|  | How about the term "buses, etc."?　（ID７） |
| ㉒ | Doing physical activity in the places you usually pass |
|  | It is the use of local resources. (ID6) |
|  | It's a little small to just walk through at the location you're using.（ID4） |
|  | Is it a little confusing to say the usual places that we usually pass through?（ID8） |
|  | What about "I usually use stairs, slopes, etc. for physical activity"?（ID8） |
